# Auditory-cued terminal knee extension enhances vastus medialis motor unit function in the early phase following ACL reconstruction: a randomized controlled trial

**DOI:** 10.1186/s43019-026-00343-3

**Published:** 2026-08-14

**Authors:** Sakda Nitkotom, Pisit Lertwanich, Pongthanayos Kiratisin, Poramate Suntornon, Ainthira Sonsukong, Patitta Rangsiyanon, Apicha Tosapornsupaluck, Roongthiwa Vachalathiti, Kantheera Areerak, Tananthorn Piamthipmanas, Kornkit Chaijenkij, Komsak Sinsurin

**Affiliations:** 1 Biomechanics and Sports Research Unit, Faculty of Physical Therapy, Mahidol University, Nakhon Pathom, Thailand; 2 Department of Rehabilitation, Pakchongnana Hospital, Nakhon Ratchasrima, Thailand; 3 Department of Orthopaedic Surgery, Faculty of Medicine Siriraj Hospital, Mahidol University, Bangkok, Thailand; 4 Faculty of Physical Therapy, Dhurakij Pundit University, Bangkok, Thailand; 5 College of Sports Science and Technology, Mahidol University, Nakhon Pathom, Thailand; 6 Musculoskeletal Physical Therapy Research Unit, Faculty of Physical Therapy, Mahidol University, Nakhon Pathom, Thailand; 7 Golden Jubilee Medical Center, Faculty of Medicine Siriraj Hospital, Mahidol University, Nakhon Pathom, Thailand

**Keywords:** Motor-sensory integration, Neuromuscular exercise, Control group, Action potential, Electromyography

## Abstract

**Background:**

Following anterior cruciate ligament reconstruction (ACLR), the vastus medialis (VM) commonly exhibits functional deficits largely attributed to neuromuscular alterations. This study aimed to investigate the immediate effects of terminal knee extension (TKE) exercise synchronized with auditory cues on VM motor unit behavior in individuals 2 weeks postACLR.

**Methods:**

The study recruited 38 individuals who underwent primary ACLR 2 weeks postsurgery and they were randomly assigned to experimental or control groups. The experimental group performed three sets of TKE exercises synchronized with auditory cues; the control group performed identical exercises without auditory cues. Before and after the intervention, VM muscle activity, motor unit action potential (MUAP), and motor unit firing rate (MUFR) were assessed during knee extension using a Trigno–Galileo electromyography system. Within and between-group differences were analyzed using parametric or nonparametric methods, as appropriate, on the basis of data distribution. Statistical significance was set at *p* < 0.05.

**Results:**

Data from 20 participants (10 experimental, 10 control) were included in the final analysis. The auditory-cued group demonstrated significantly greater increases in peak [12.59 (IQR 17.89) µV, *p* = 0.043] and average [11.31 (IQR 3.04) µV, *p* = 0.029] MUAP compared with the noncued group [6.26 (IQR 11.02) µV and 2.24 (IQR 9.94) µV, respectively]. Moreover, only the auditory-cued group showed significant postintervention increases in average electromyography (EMG) activity [pre: 14.23 (IQR 11.37) µV; post: 17.60 (IQR 20.94) µV; *p* = 0.007], average MUAP [pre: 43.07 (IQR 34.30) µV; post: 50.33 (IQR 36.12) µV; *p* = 0.005], peak MUAP [pre: 55.54 (IQR 50.35) µV; post: 61.69 (IQR 52.92) µV; *p* = 0.005], and peak MUFR (pre: 10.50 ± 4.29 pps; post: 12.66 ± 3.36 pps; *p* = 0.016) after the intervention.

**Conclusions:**

A single session of TKE exercise synchronized with auditory cues significantly increased MUAP in the VM muscle in individuals 2 weeks post-ACLR. These findings likely reflect enhanced acute neural drive and improved VM motor unit recruitment. Auditory-cued TKE may represent a potential strategy to enhance neuromuscular control during the early postoperative phase. However, these effects reflect only immediate responses, and the persistence of these adaptations with longer-term training remains to be determined.

Trial registration: NCT06662955 (25 October 2024, on ClinicalTrials.gov).

## Background

Anterior cruciate ligament (ACL) injury is a common knee injury among athletes, with particularly high incidence rates in sports that involve cutting, pivoting, and jumping movements, such as American football, basketball, soccer, and volleyball [1, 2]. Anterior cruciate ligament reconstruction (ACLR) is considered the standard procedure for restoring knee stability and enabling return to sport [3]. However, this intervention imposes a substantial economic burden, with the total costs of surgery and rehabilitation exceeding US $38,000 [4]. Despite surgical treatment, approximately 45% of individuals undergoing ACLR are unable to return to competitive sport [5]. In addition, the timeline for returning to sport is prolonged, with most athletes requiring approximately 7–12 months to resume participation [6, 7].

A critical concern following ACLR is quadriceps muscle impairment, particularly deficits in the vastus medialis (VM) muscle [8, 9]. Quadriceps functional deficits are detectable in the early postoperative phase and are associated with a 15–40% reduction in muscle strength compared with preoperative levels, which may persist after surgery [8, 9]. The VM muscle plays a crucial role in maintaining knee stability and is essential for athletic performance, particularly during activities requiring high running speeds and explosive movements such as jumping [10, 11]. Although various interventions, including strengthening exercises, neuromuscular training, and electrical stimulation, have been used to address quadriceps dysfunction [12], persistent weakness remains prevalent, with up to 40% of individuals demonstrating strength deficits 3 years after surgery [9]. These deficits must be addressed to optimize rehabilitation strategies and improve clinical outcomes.

Arthrogenic muscle inhibition has been proposed as a key mechanism underlying persistent quadriceps weakness following ACLR [13]. Joint pain, inflammation, and effusion are prominent in the early phase after injury and may disrupt afferent sensory input, leading to altered spinal reflex activity and asymmetrical cortical excitability between hemispheres within the first 2 weeks after ACLR [13–16]. These alterations may reduce motor output and impair the motor unit behavior required for effective muscle contraction [17].

Therapeutic exercise interventions targeting both muscular and neural functions may help address neuromuscular deficits of the VM in individuals with ACLR. The incorporation of auditory cues, such as metronome beats, has been employed in sports training to enhance performance in activities including hammer throwing [18], soccer [19], and hurdling [20]. Moreover, incorporating a metronome during resistance training has been shown to increase motor cortex excitability more effectively than self-paced training [21, 22]. Given the established relationship between cortical excitability and motor unit recruitment, therapeutic exercise combined with auditory cues may be a promising strategy to enhance neuromuscular function. This approach may be particularly relevant during rehabilitation following ACLR, where optimizing the neural drive is critical for recovery and functional performance.

This study aimed to investigate the effects of therapeutic exercise with auditory cues on motor unit behavior following ACLR. Terminal knee extension (TKE) is a well-established exercise commonly used in knee rehabilitation [23]. As a closed kinetic chain exercise, TKE preferentially activates the VM muscle and is considered a safe and effective approach for facilitating quadriceps activation during the early postoperative period following ACLR [24, 25]. Furthermore, auditory-cued TKE has demonstrated the potential to preserve motor unit action potential (MUAP) characteristics. However, previous studies have been conducted predominantly in healthy individuals [26]. Therefore, it remains unclear whether the neuromuscular effects of auditory-cued TKE can be translated to individuals following ACLR, specifically during the early postoperative period when neural activation deficits are most pronounced. To the best of our knowledge, this was the first study to investigate the immediate effects of a single session of auditory-cued TKE on VM motor unit behavior in individuals with ACLR, particularly within the first 2 weeks post-surgery. We hypothesized that incorporating auditory pacing would enhance neuromuscular control by modulating motor unit behavior in the VM muscle immediately after the intervention. This study extends previous TKE-decomposition electromyography (dEMG) findings from healthy individuals to a clinically relevant postoperative population.

## Methods

### Study design

This randomized controlled trial was conducted in a clinical setting.

### Participants

**Fig. 1:**
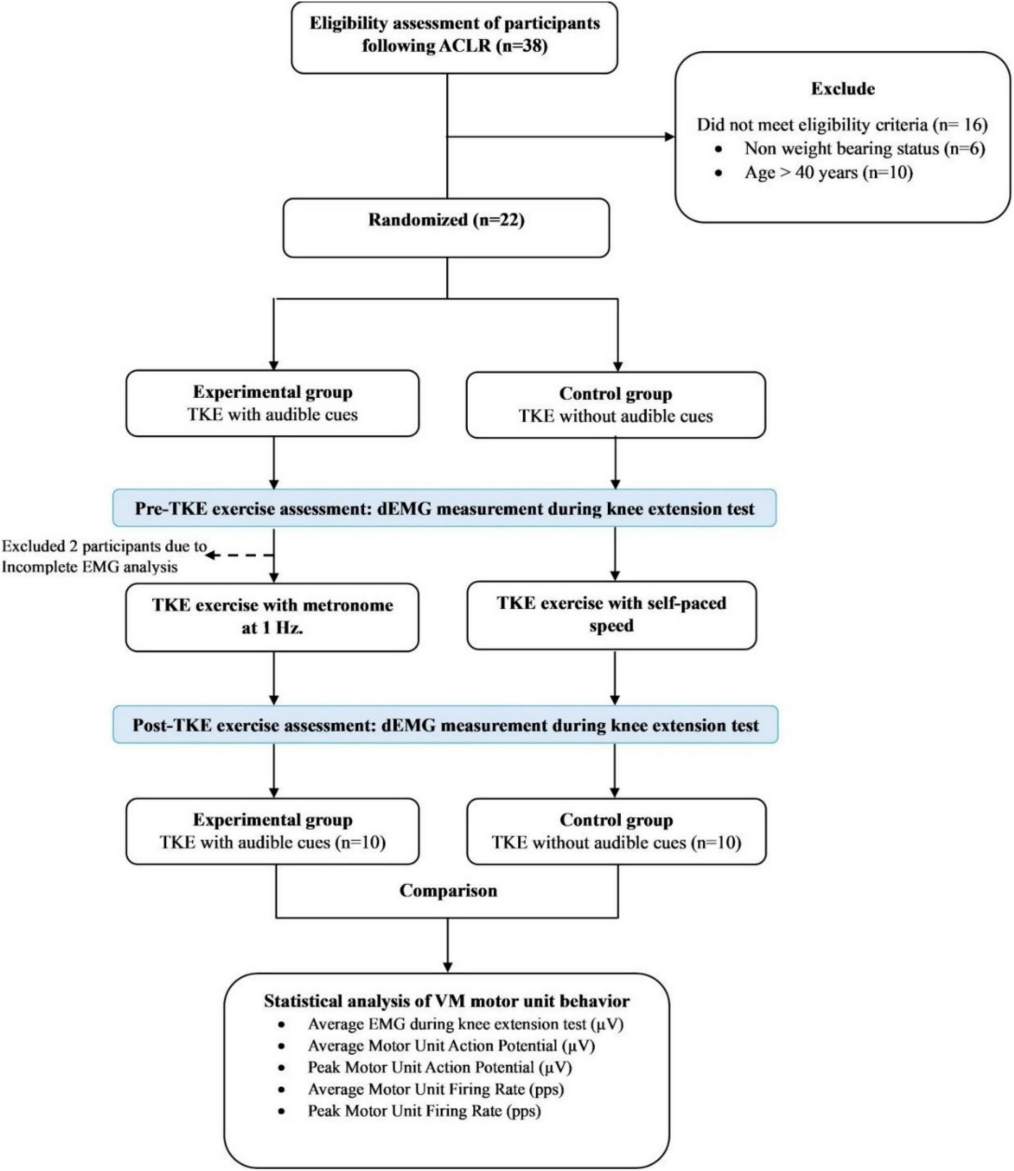
Consolidated Standards of Reporting Trials (CONSORT) flow diagram illustrating participant enrollment and analysis

Individuals who sustained ACLR injury during sports activities and subsequently underwent ACLR were invited to participate and screened for eligibility (Fig. 1). Participants who had undergone primary ACLR within 2 weeks post-surgery were recruited between November 2024 and May 2025. Recruitment was conducted at the Faculty of Physical Therapy, Siriraj Hospital, and the Golden Jubilee Medical Center, Mahidol University. Eligible participants were male or female athletes aged 18–40 years with a preinjury Tegner activity scale score > 4 and who had received either a hamstring tendon autograft or bone–patellar tendon–bone autograft. Individuals were excluded if they had a nonweight-bearing status (e.g., owing to meniscus or cartilage repair), limited knee extension range of motion, an inability to achieve > 30° of active or passive knee flexion in the operated limb, or a history of severe lower extremity injury or musculoskeletal disorders (e.g., fracture) within the past 6 months.

The sample size was calculated using G^*^Power (version 3.1.9.4). On the basis of a repeated-measures analysis of variance examining the within–between interaction, a total sample of 20 participants was required to achieve 80% statistical power at an alpha level of 0.05. The calculation assumed a large effect size (partial eta squared = 0.14) on the basis of a previous study investigating similar motor unit behavior outcomes [26] and included an anticipated dropout rate of 10%. Participants were randomly allocated to two groups using a lottery method: the experimental group performed TKE exercises synchronized with auditory cues using a metronome (1 Hz) [26], whereas the control group performed TKE exercises without auditory cues at a self-selected pace. To ensure baseline comparability, matched pairs were used to guide balance across groups and were formed on the basis of age (± 3 years), sex, graft type, presence of meniscus surgery, Tegner activity scale level, and dominant leg. All participants provided written informed consent before participation.

### Study procedure

**Fig. 2:**
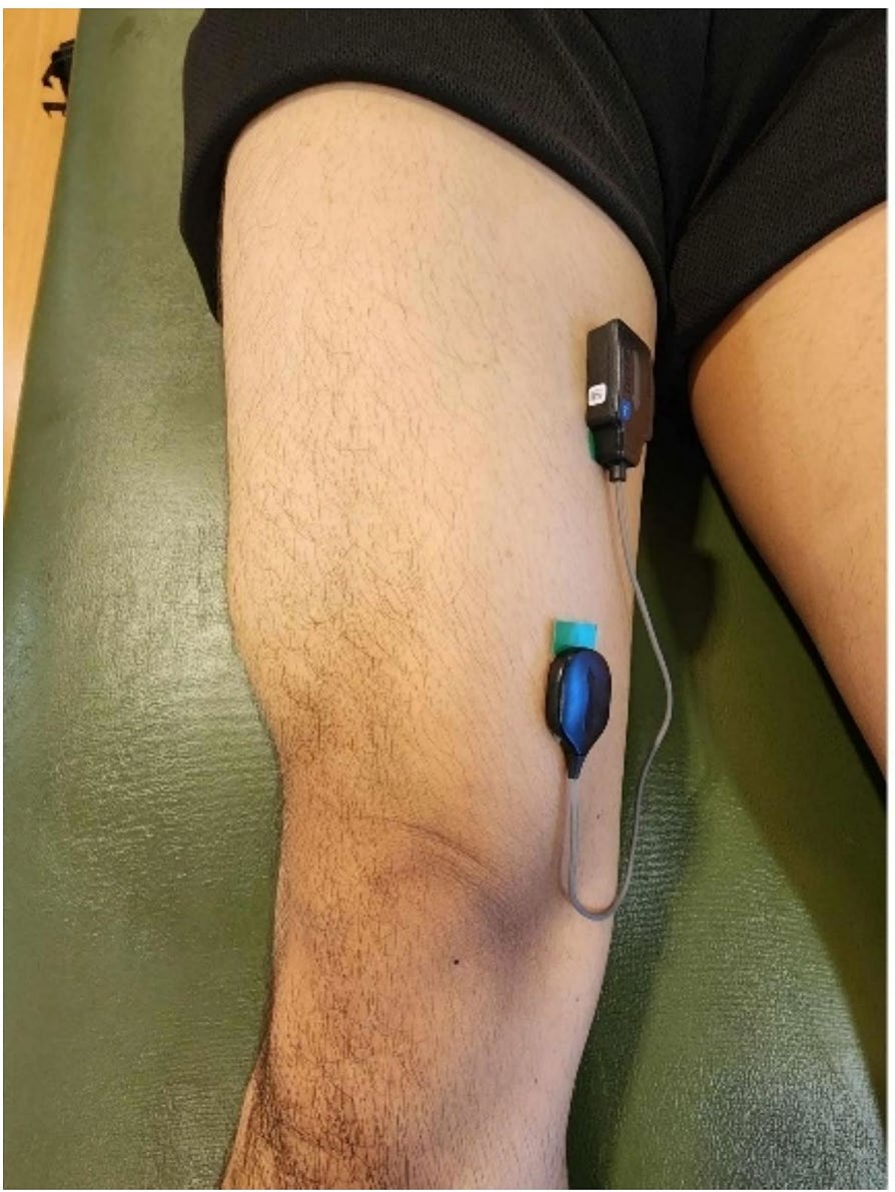
EMG sensor placement for the VM muscle

VM muscle activity during knee extension was assessed before and after TKE exercises. The motor unit behavior of the VM muscle was recorded using decomposition electromyography (dEMG) with four-channel Trigno–Galileo sensors (Delsys Inc., Boston, MA, USA) [27]. The system provides a 16-bit resolution with a bandwidth of 20–450 Hz, and EMG signals were sampled at 1000 Hz using EMGworks 4.0 software (Delsys Inc.). Sensors were placed on the operated thigh at 80% of the distance along the line between the anterior superior iliac spine and the joint space at the anterior border of the medial collateral ligament of the knee, in accordance with surface electromyography for the noninvasive assessment of muscles (SENIAM) guidelines [28] (Fig. 2). Hair at the sensor site was shaved, and the skin was cleaned with alcohol to reduce impedance. Before EMG data acquisition, signal quality was verified during active knee extension in a seated position.

A baseline noise level < 10 µV and a green-zone status on the system quality meter were required [25, 28].

### Knee extension tests before and after TKE exercise

**Fig. 3:**
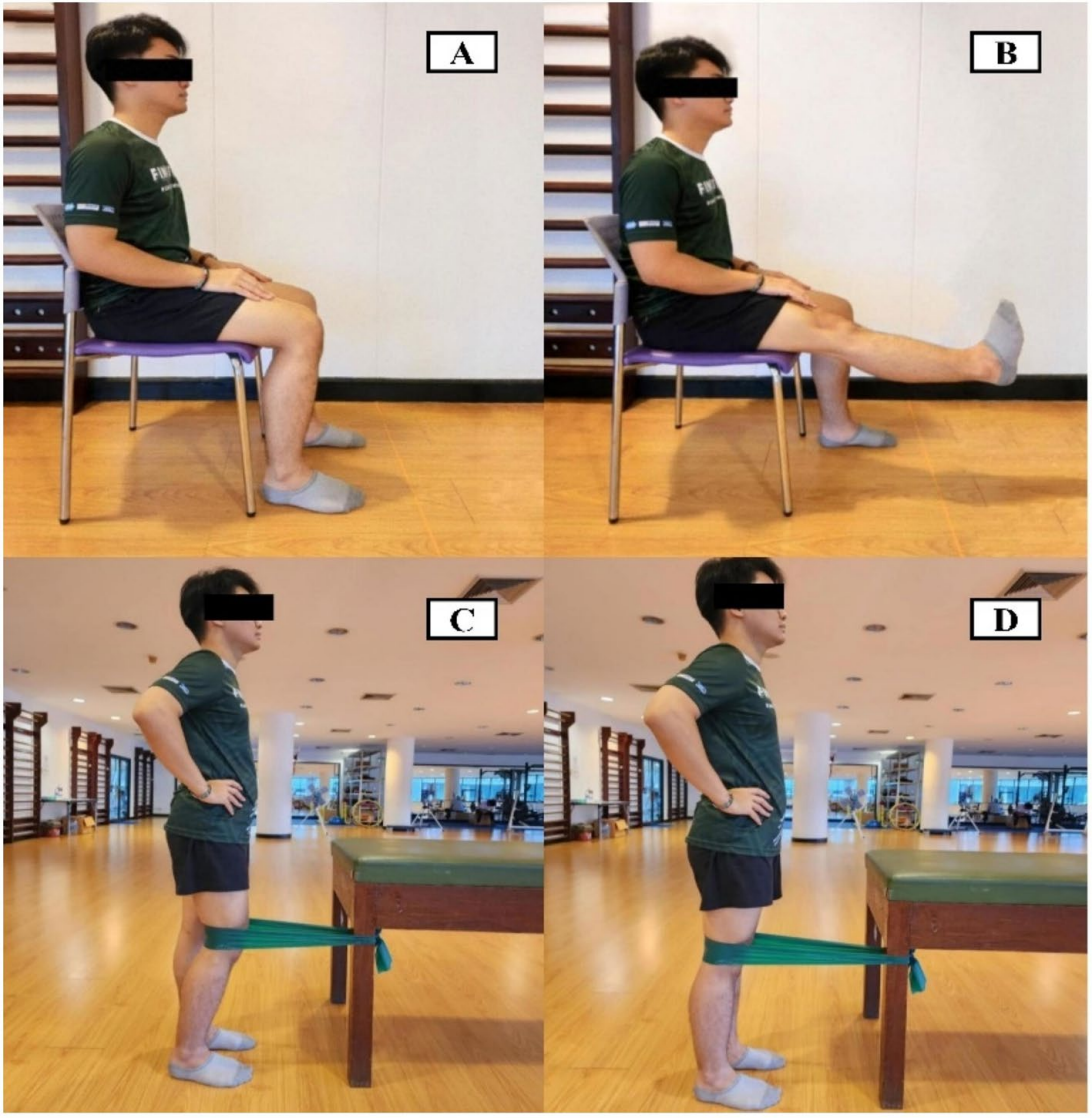
Example of the knee extension test (A, B) and the terminal knee extension exercise (C, D)

Participants were instructed to perform five repetitions of knee extension (Fig. 3A, B). Each contraction was held for 5 s and separated by 5 s of rest, resulting in a total task duration of 45 s. Participants then completed a 10 min seated rest period to minimize the influence of acute fatigue before the TKE intervention.

### TKE exercise

Participants began in a standing position with their feet shoulder-width apart (Fig. 3C, D). An elastic resistance band (TheraBand^®^, green, 2.1 kg resistance; The Hygenic Corporation, Akron, OH, USA) was placed around the knee during the exercise. Training intensity was monitored using the Borg category-ratio scale (0–10) [29], with a target intensity of 6–7, corresponding to a “very intense” level and approximately 70% of one-repetition maximum [30].

In the auditory-cued TKE group, participants synchronized knee flexion and extension with metronome cues at 1 Hz [24]. Before the intervention, all participants were instructed on proper TKE technique, and a physical therapist assessed movement quality to ensure safety and readiness. The exercise consisted of 10 repetitions per set for a total of three sets, with 3 min rest intervals between sets. Pain or discomfort during exercise and rest was monitored according to the optimal loading concept, with acceptable pain levels ranging from 0 to 5 on the numeric rating scale [31]. In the control group, participants performed the same TKE protocol but at a self-selected pace without auditory cues.

### EMG data acquisition

**Fig. 4:**
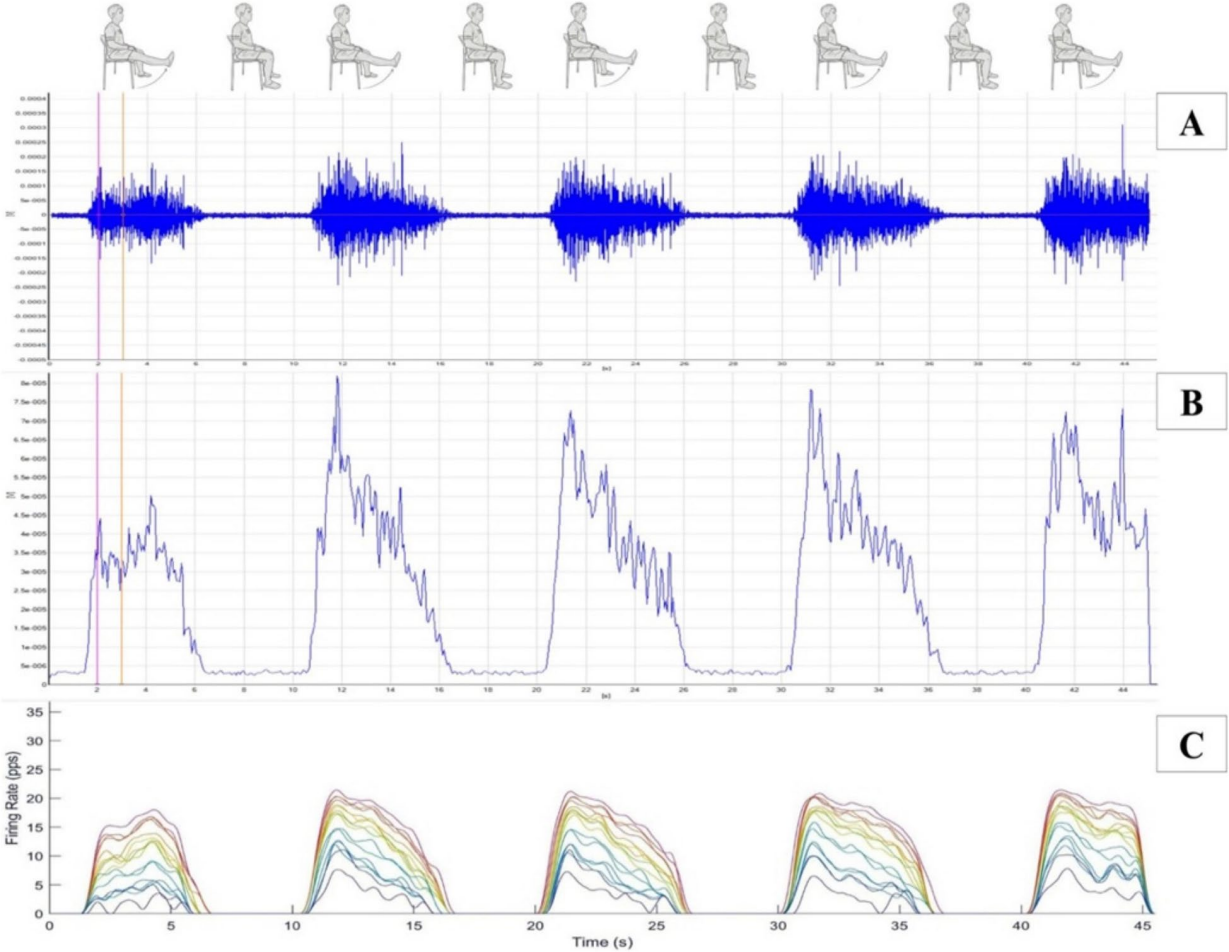
Example of VM EMG data during knee extension tests. (A) Raw EMG signal. (B) Filtered EMG signal. (C) dEMG results illustrating motor unit analysis

The motor unit behavior of the VM muscle was the primary outcome of the present study. EMG signals were decomposed and analyzed using NeuroMap software (version 1.1.0; Delsys Inc., Boston, MA, USA). The software algorithm identifies and characterizes motor unit activity by processing EMG signals and isolating individual motor unit firing patterns (Fig. 4C). It further visualizes motor unit behavior, including firing rates and action potentials. Average and peak MUAPs and MUFRs were calculated and compared between the groups.

Average EMG was the secondary outcome of the present study. VM muscle activity was assessed using average EMG data obtained during knee extension tests and processed offline using EMGworks Analysis software (version 4.0; Delsys Inc., USA). Signals from channel 1 were analyzed. Raw EMG signals were processed using a fourth-order band-pass Butterworth filter (20–450 Hz), followed by full-wave rectification and smoothing using a moving root mean square window of 50 ms (Fig. 4B). Changes in average EMG following TKE exercise were calculated and compared between the groups. Average EMG was computed over a time window spanning 10 ms before and after the peak EMG amplitude of the third repetition during the knee extension test.

### Statistical analysis

Statistical analyses were performed using the statistical package for the social sciences software (IBM, version 31). Data normality was assessed using the Shapiro–Wilk test. For normally distributed data, paired *t*-tests and independent *t*-tests were used to evaluate within-group and between-group differences, respectively. For non-normally distributed data, the Wilcoxon signed-rank test and Mann–Whitney *U* test were applied. A *p*-value < 0.05 was considered statistically significant.

## Results

Of the athletes, 38 who underwent ACLR within the first 2 weeks postsurgery were initially recruited (Fig. 1). Of the athletes, 16 were excluded on the basis of the predefined exclusion criteria, and EMG data from two additional athletes were incomplete and therefore excluded from the analysis. Consequently, data from 20 participants (10 experimental, 10 control) were included in the final analysis. Participant characteristics are presented in Table 1 and showed no significant differences between groups (*p* > 0.05).

**Table 1:** Comparison of participant characteristics between groups

Demographics	TKE with audible cues (*n* = 10)	TKE without audible cues (*n* = 10)	*p*-value
	Mean ± SD	Mean ± SD	
Age (years)	28.3 ± 4.9	27.5 ± 6.3	0.75^b^
BMI (kg/m^2^)	24.6 ± 4.0	24.6 ± 2.9	0.97^b^
Tegner activity scale (preinjury)	6.8 ± 1.8	7.3 ± 2.0	0.58^c^
Time to surgery (months)	3.7 ± 4.0	3.8 ± 3.2	0.53^c^
Gender (female/male)	2/8	2/8	0.48^a^
Graft type (BPTB/HS)	5/5	4/6	0.48^a^
Surgery side (right/left)	5/5	6/4	0.48^a^
Dominant leg (right/left)	10/0	9/1	0.95^a^
Meniscectomy (yes/no)	2/8	1/9	0.48^a^

BMI, body mass index; BPTB, bone–patellar tendon–bone; HS, hamstring tendon

^a^tested with the chi-squared test

^b^tested with the independent *t*-test

^c^tested with the Mann–Whitney *U* test

Table 2 summarizes within-group changes in motor unit behavior before and after the intervention. The auditory-cued group demonstrated significant increases in average EMG [pre: 14.23 (IQR 11.37) µV; post: 17.60 (IQR 20.94) µV; *p* = 0.007], average MUAP [pre: 43.07 (IQR 34.30) µV; post: 50.33 (IQR 36.12) µV; *p* = 0.005], peak MUAP [pre: 55.54 (IQR 50.35) µV; post: 61.69 (IQR 52.92) µV; *p* = 0.005], and peak MUFR (pre: 10.50 ± 4.29 pps; post: 12.66 ± 3.36 pps; *p* = 0.016) following the intervention. In contrast, the control group (TKE without auditory cues) showed no significant pre–post differences in any outcome measure.

**Table 2:** Within-group comparisons of average EMG, MUAP, and MUFR

	TKE with audible cues (*n* = 10)			TKE without audible cues (*n* = 10)		
	Mean ± SD	Median	IQR	Mean ± SD	Median	IQR
	“Average MUAP (µV)”					
Pretraining	54.91 ± 50.85	43.07	34.30	35.42 ± 16.27	35.40	25.62
Post-training	68.84 ± 60.00	50.33	36.12	40.22 ± 12.29	37.79	14.49
*p*-value within group	^*^0.005^b^			0.295^a^		
Effect size (Cohen’s *d*)	0.890			0.350		
	“Peak MUAP (µV)”					
Pretraining	79.22 ± 82.26	55.54	50.35	47.41 ± 22.80	49.89	37.47
Post-training	94.94 ± 88.12	61.69	52.92	54.64 ± 16.87	51.70	22.76
*p*-value within group	^*^0.005^b^			0.264^a^		
Effect size (Cohen’s *d*)	0.890			0.380		
	“Average MUFR (pps)”					
Pretraining	3.92 ± 1.11	4.29	1.86	3.37 ± 1.45	3.83	1.65
Post-training	4.26 ± 1.25	4.18	2.32	3.60 ± 1.23	3.80	1.19
*p*-value within group	0.350^a^			0.445^b^		
Effect size (Cohen’s *d*)	0.034			0.240		
	“Peak MUFR (pps)”					
Pretraining	10.50 ± 4.29	10.87	4.56	9.40 ± 3.72	10.41	2.71
Post-training	12.66 ± 3.36	11.86	5.46	11.60 ± 3.00	12.37	3.58
*p*-value within group	^*^0.016^a^			0.074^b^		
Effect size (Cohen’s *d*)	0.940			0.560		
	“Average EMG during knee extension test (µV)”					
Pretraining	24.90 ± 35.54	14.23	11.37	14.91 ± 7.81	12.23	11.44
Post-training	32.54 ± 43.88	17.60	20.94	16.51 ± 7.87	19.99	11.60
*p*-value within group	^*^0.007^b^			0.353^a^		
Effect size (Cohen’s *d*)	0.850			0.310		

CI, confidence interval; SD, standard deviation; IQR, interquartile range

^a^tested with the paired *t*-test

^b^tested with the Wilcoxon signed-rank test

Table 3 presents between-group differences in mean change scores following the TKE exercise. The auditory-cued group exhibited significantly greater increases in average and peak MUAP compared with the control group [11.31 (IQR 3.04) µV and 12.59 (IQR 17.89) µV] than the control group [2.24 (IQR 9.94) µV and 6.26 (IQR 11.02) µV, respectively]. No significant between-group differences were observed for MUFR and average EMG.

**Table 3:** Between-group comparisons of mean changes in average EMG, MUAP, and MUFR

Parameter	Group	Mean difference					Effect size	
		Mean ± SD	95% CI (lower, upper)	Median	IQR	*p*-value	Cohen’s *d*	95% CI (lower, upper)
Average MUAP (µV)	Audible cues	13.93 ± 10.43	(6.47, 21.39)	11.31	3.04	^*^0.029^b^	0.75	−0.16, 1.66
	Without audible cues	4.80 ± 13.65	(−4.96, 14.57)	2.24	9.94			
Peak MUAP (µV)	Audible cues	15.73 ± 10.03	(8.55, 22.90)	12.59	17.89	^*^0.043^b^	0.54	−0.35, 1.42
	Without audible cues	7.22 ± 19.17	(−6.49, 20.94)	6.26	11.02			
Average MUFR (pps)	Audible cues	0.34 ± 1.09	(−0.44, 1.12)	0.59	0.97	0.880^a^	0.06	−0.82, 0.94
	Without audible cues	0.23 ± 2.13	(−1.30, 1.75)	0.22	1.62			
Peak MUFR (pps)	Audible cues	2.15 ± 2.29	(0.52, 3.79)	1.68	1.45	0.853^b^	−0.01	−0.89, 0.87
	Without audible cues	2.20 ± 4.40	(−0.94, 5.35)	1.73	3.04			
Average EMG during knee extension test (µV)	Audible cues	7.65 ± 9.21	(1.06, 14.23)	3.44	8.74	0.190^b^	0.80	−0.12, 1.72
	Without audible cues	1.60 ± 5.16	(−2.09, 5.29)	2.49	9.58			

CI, confidence interval; SD, standard deviation; IQR, interquartile range

^a^tested with the independent *t*-test

^b^tested with the Mann–Whitney *U* test

## Discussion

The aim of the present study was to investigate the immediate effects of TKE exercise synchronized with auditory cues on motor unit behavior in the VM muscle in individuals 2 weeks postACLR. The MUAP, MUFR, and average EMG of the VM muscle were compared before and after a single TKE session. In addition, mean changes in all parameters were compared between the auditory-cued and noncued groups. Participant characteristics did not differ significantly between groups, including sex, age, BMI, graft type, dominant leg, and meniscectomy status (Table 1).

### MUAP

Exercise-induced alterations in MUAP characteristics suggest neuromuscular adaptation, particularly under synchronized training conditions [26]. The present findings indicate that following TKE exercise, average EMG and MUAP of the VM muscle increased (Table 2). However, only the auditory-cued group demonstrated significant increases in average EMG (*p* = 0.007), peak MUAP (*p* = 0.005), and average MUAP (*p* = 0.005) after the intervention. These findings indicate a potential advantage of TKE exercise with auditory cueing in individuals with ACLR, particularly in the early phase of rehabilitation. The present findings supported our previous study in healthy individuals [26], which demonstrated that auditory-cued TKE preserved the average and peak MUAP amplitudes before (54.91 ± 50.85 and 79.22 ± 82.26 µV, respectively) and after exercise (68.84 ± 60.00 and 94.94 ± 88.12 µV, respectively). Similarly, in the present study, significantly greater increases in average and peak MUAP amplitudes following TKE were observed in the auditory-cued group (13.93 ± 10.43 and 15.73 ± 10.03 µV, respectively) compared with the control group (4.80 ± 13.65 and 7.22 ± 19.17 µV, respectively) (Table 3). Collectively, these findings suggest that auditory cueing may facilitate motor unit recruitment and neuromuscular activation across healthy and postoperative populations. The observed increases in MUAP amplitude likely reflect the recruitment of larger motor units in response to increased task demands [32]. Larger motor units, which innervate a greater number of muscle fibers, generate higher-amplitude action potentials, and their preferential recruitment under higher force requirements is consistent with established motor unit recruitment principles [33]. Accordingly, the increased MUAP amplitude observed following auditory-cued TKE exercise may serve as an indirect indicator of greater recruitment of larger motor units. Synchronized movement execution with a metronome likely imposes greater demands on the central nervous system to generate precise, temporally coordinated motor commands [34]. Auditory cues may enhance movement timing and neural modulation through auditory–motor coupling mechanisms involving a distributed neural network, including the auditory cortex, primary motor cortex (M1), sensorimotor cortex, premotor cortex, supplementary motor area, and cerebellum [35, 36]. The integration of auditory and motor information may facilitate motor planning and temporal coordination, thereby influencing motor unit recruitment during exercise. Therefore, TKE exercise combined with auditory cues may modulate motor unit behavior through enhanced neural drive compared with TKE exercise alone.

During group comparisons, greater increases in average (*p* = 0.029) and peak MUAP (*p* = 0.043) were observed in the auditory-cued group compared with the noncued TKE group (Table 3), indicating a more pronounced enhancement of VM motor unit behavior with auditory cueing. However, no significant between-group difference in average EMG was observed. Although surface EMG provides a global representation of muscle activation, it offers limited insight into individual motor unit behavior. In contrast, advanced EMG techniques incorporating motor unit decomposition allow for a more detailed evaluation of motor unit characteristics and provide greater sensitivity for detecting exercise-induced neural adaptations, particularly at the motor unit level in response to acute interventions.

### MUFR

Resistive exercise induces neural adaptations that contribute to increased force production, specifically during the early phase of training [37, 38]. Motor units are recruited in an orderly sequence, progressing from low-threshold, slow-twitch units to higher-threshold, fast-twitch units as force demands increase. In addition to recruitment strategies, increases in MUFR reflect enhanced rate coding, which contributes to greater force output through increased motor neuron discharge frequency [37]. The present findings demonstrated increases in average and peak MUFR in both groups (Table 2). However, a marked increase in peak MUFR was observed only in the auditory-cued group, increasing from 10.50 ± 4.29 pps at baseline to 12.66 ± 3.36 pps postintervention (*p* = 0.016). In contrast, no marked within-group change in peak MUFR was observed in the noncued TKE group (*p* = 0.074), and no significant between-group differences were detected (*p* = 0.853).

Changes in MUFR observed in both groups may reflect increased neural drive. The motor neuron pool is regulated through coordinated modulation of motor unit recruitment and discharge rates in response to increasing force demands, consistent with the size principle of motor unit recruitment [39]. Notably, a significant increase in peak MUFR was observed only in the auditory-cued TKE group (*p* = 0.016) (Table 2), suggesting that externally paced auditory input provides an additional facilitatory effect on motor unit firing behavior beyond exercise alone. Peak MUFR may be particularly sensitive to changes in maximal neural drive, which may explain the selective enhancement observed under the auditory-cued condition. Auditory cues likely enhance sensorimotor integration by improving attentional focus during movement execution, thereby augmenting corticospinal excitability and descending motor commands [34, 40]. This enhanced neural input may enable recruited motor units to achieve higher peak MUFR.

### Clinical implications, study limitations, and future directions

The findings of the present study indicate that TKE exercise may enhance neuromuscular control through MUAP and MUFR responses. Moreover, TKE exercise synchronized with auditory cues elicited greater improvements in both MUAP and MUFR compared with exercise performed without auditory cues. Synchronization between movement and auditory rhythm appears to be a key component of the intervention. Therefore, TKE exercise combined with auditory cues may represent a potential intervention for individuals who have undergone ACLR and experience quadriceps impairment, particularly VM deficits. This approach may enhance neural drive and efferent signal transmission to the VM muscle, thereby promoting motor unit recruitment during early stages of rehabilitation. In addition, no adverse effects were reported, supporting the concept of optimal loading following ACLR. Clinically, this intervention may improve knee control during the stance phase of gait and potentially accelerate recovery compared with conventional rehabilitation approaches; however, these potential benefits require further investigation.

However, the present findings reflect only the immediate responses to a single TKE exercise session, and the long-term persistence of these neural adaptations remains unknown. In addition, the relatively small sample size, the presence of arthrogenic muscle inhibition (AMI) symptoms, and the clinical significance of the observed change should be considered when interpreting the results. Furthermore, the absence of functional outcome measures limits the ability to determine whether the observed neuromuscular improvements translate into meaningful functional benefits.

Future studies incorporating larger cohorts, comprehensive assessments of AMI-related symptoms, functional outcome measures, long-term follow up, and comparisons with other AMI-targeted interventions are warranted to validate and extend the present findings.

## Conclusions

Significantly greater peak and average MUAP were observed in the auditory-cued group than in the noncued group. Following a session of TKE exercises synchronized with auditory cues, significant increases in MUAP were observed in the VM muscle of individuals within the first 2 weeks post-ACLR. These improvements likely reflect enhanced neural drive and acute neuromuscular changes in VM motor unit behavior. Collectively, these findings suggest that auditory-cued TKE exercise may represent a potential intervention for enhancing motor unit recruitment and neuromuscular control during the early postoperative phase.

## Data Availability

The datasets used and/or analyzed during the current study are available from the corresponding author on reasonable request.

## Abbreviations

ACL: Anterior cruciate ligament
ACLR: Anterior cruciate ligament reconstruction
MUAP: Motor unit action potential
MUFR: Motor unit firing rate
RPE: Rating of perceived exertion
VM: Vastus medialis
